# Bonobo personality predicts friendship

**DOI:** 10.1038/s41598-019-55884-3

**Published:** 2019-12-17

**Authors:** Jonas Verspeek, Nicky Staes, Edwin J. C. van Leeuwen, Marcel Eens, Jeroen M. G. Stevens

**Affiliations:** 1 Behavioural Ecology and Ecophysiology Group, Department of Biology, University of Antwerp, Antwerp, Belgium; 2 Centre for Research and Conservation, Royal Zoological Society of Antwerp, Antwerp, K. Astridplein 26, B 2018 Antwerp, Belgium; 3 Centre for the Advanced Study of Human Paleobiology; Department of Anthropology, The George Washington University, Washington, DC USA; 4 Max Planck Institute for Psycholinguistics, Wundtlaan 1, 6525 XD Nijmegen, The Netherlands

**Keywords:** Behavioural ecology, Animal behaviour

## Abstract

In bonobos, strong bonds have been documented between unrelated females and between mothers and their adult sons, which can have important fitness benefits. Often age, sex or kinship similarity have been used to explain social bond strength variation. Recent studies in other species also stress the importance of personality, but this relationship remains to be investigated in bonobos. We used behavioral observations on 39 adult and adolescent bonobos housed in 5 European zoos to study the role of personality similarity in dyadic relationship quality. Dimension reduction analyses on individual and dyadic behavioral scores revealed multidimensional personality (Sociability, Openness, Boldness, Activity) and relationship quality components (value, compatibility). We show that, aside from relatedness and sex combination of the dyad, relationship quality is also associated with personality similarity of both partners. While similarity in Sociability resulted in higher relationship values, lower relationship compatibility was found between bonobos with similar Activity scores. The results of this study expand our understanding of the mechanisms underlying social bond formation in anthropoid apes. In addition, we suggest that future studies in closely related species like chimpanzees should implement identical methods for assessing bond strength to shed further light on the evolution of this phenomenon.

## Introduction

Populations of group-living species comprise individuals who differ in the level of interaction they have with others^1,2^. These interactions occur non-randomly and often result in lasting and stable social bonds, also called friendships^3^, that can improve individual fitness^4^. For example, offspring survival is higher in social female yellow baboons^5^ and in feral horses with more and stronger female-male bonds^6^. Similarly, in marmosets, breeding pairs and breeder-helper dyads with stronger bonds contributed more in offspring care^7^. In bottlenose dolphins, strong bonds between males increased mating chance^8^, while male-female bonding increased the lifespan of juvenile males^9^. Spotted hyenas^10^, chimpanzees^11^ and also humans^12^ engage in more cooperative interactions with friends. Often age, sex, kinship or rank similarity are used to explain variation in the strength of social bonds^5,13–15^. However, the influence of these factors is very inconsistent across studies and often species-specific. For example, in chimpanzees strong social bonds are typically formed between related dyads and individuals of similar age, but are also found between unrelated non-age-mates^16–18^. Social living animals tend to associate with similar individuals^19–22^, a phenomenon called homophily^23^. In humans homophily has been found across many phenotypic dimensions like sex, age and class^23–25^ but also personality^26–28^. Recently, homophily in personality has been studied as an important factor contributing to the existing variability in social relationships across a range of phylogenetically distant taxa. For example, similarity in exploratory behavior influenced assortment in female zebra finches^29^ and male great tits^30^, similarity in Boldness predicted bonding in baboons^20^ and Sociability scores predicted relationship quality in chimpanzees^21^ and capuchin monkeys^31^.

While several studies have used relatively simple measures to assess relationship quality, including only one or just a few behaviors like agonistic support^32^, proximity^21,30^ or time spent in proximity and grooming^20,33,34^, a more inclusive way of determining relationship quality is to use composite measures. Cords and Aureli^35^ introduced a composite model to measure relationship quality consisting of following three components: value, compatibility and security. The *value* of a relationship comprises the benefits that result from that relationship like food sharing or agonistic support. The *compatibility* between two partners is a measure of the level of tolerance between individuals, and reflects the general nature of social interactions in a dyad. This means that in dyads with frequent aggressive interactions and counter-interventions, the nature of the relationship is defined as less tolerant. The predictability and consistency of the behavior of both partners over time describes the *security* of a relationship^30,36,37^. This three-component model has already been implemented in different study species. Relationship value contained behaviors relating to mainly social affiliation, tolerance and support in chimpanzees^36,38^, ravens^39^, Japanese macaques^40^, capuchin monkeys^31^, bonobos^41^ and dolphins^42^. Relationship compatibility contained aggressive behaviors in all but two studies^40,42^ and for the third component of relationship quality, security, mixed results have been found across studies^31,33–35,37,43^. Behaviors loading on this component greatly differed, making this component the least consistent across studies.

Homophily in personality seems to be widespread among different taxa, albeit in different personality traits with varying results. Studying closely related species may help in explaining these differences and in understanding how homophily in personality evolved. While homophily in personality has been studied in both humans^27,28^ and chimpanzees^21^, no studies have been done in our other close relative, the bonobo. Bonobo societies are characterized by complex social relationships, where the strongest bonds are found between females^44–46^ and between females and their adult sons^45,47–49^. A previous study on bonobos^41^ found that relationship value was highest between unrelated female-female dyads and related male-female dyads. Relationship compatibility was highest between female-female dyads and between bonobos with large rank distances. However, not all variability in relationship quality could be explained by sex, rank, age and relatedness^41^. In this study, we aim to investigate the potential influence of personality on dyadic relationship quality. Bonobos within the same social group exhibit remarkable individual differences in personality^50,51^, and bonobos may partly choose who they want to associate with based on similarity or differences in personality. We previously identified personality in bonobos using behavioral observations, and found four personality traits: Sociability, Boldness, Openness and Activity^52^. Here, we aim to find how similarity in each of the four personality traits impacts dyadic relationship quality in bonobos. Based on previous findings in chimpanzees and capuchin monkeys^31^ we expect to find a link between similarity in Sociability and relationship value. Our study will be the first to use the composite measure for different aspects of relationship quality to report on the potential role of personality similarity on relationship compatibility.

## Results

### Relationship quality

Eight dyadic behavioral variables were included in the first exploratory factor analysis. Sampling adequacy was high (KMO = 0.652) and inter-variable correlations were sufficiently high (Bartlett’s test of sphericity: chisq = 275.284, df = 15, p < 0.001). Initial exploration using factor analysis revealed a three-component solution. However only one item, grooming symmetry, loaded on the third dimension, and therefore a new factor analysis was conducted^53^ maintaining only two factors. Next, grooming symmetry and aggression symmetry were excluded from the EFA based on low factor loadings (Table 1). Varimax- and promax-rotated dimensions did not differ substantially.

**Table 1:** Varimax rotated factor loadings for the components of Relationship Quality.

Variable	Varimax rotation	
	Value	Incompatibility
Proximity	**0.945**	−0.114
Grooming Frequency	**0.834**	−0.025
Peering	**0.579**	−0.058
Support	**0.436**	0.062
Counter-intervention	0.058	**0.709**
Aggression frequency	−0.084	**0.595**
Eigenvalue	2.49	1.42
% of variation explained	0.36	0.14

Boldface highlights loadings > |0.4|.

The first factor explained 36% of the total variance and had positive loadings for proximity, grooming frequency, peering, and support. This component is very similar to the relationship value component of previous studies, and included traits related to fitness such as coalitionary support^35,36,41^ and was thus labelled “value”. The second component explained 14% of the total variance and had positive loadings for counter-intervention and aggression frequency and thus was similar to the second component found by previous studies^36,41^, “relationship incompatibility”. To make this factor easier for further interpretation, we reversed the signs for this component and relabeled it “compatibility”^36,41^.

### The influence of genetic sex combination and similarity in personality on relationship quality

#### Relationship value

**Figure 1:**
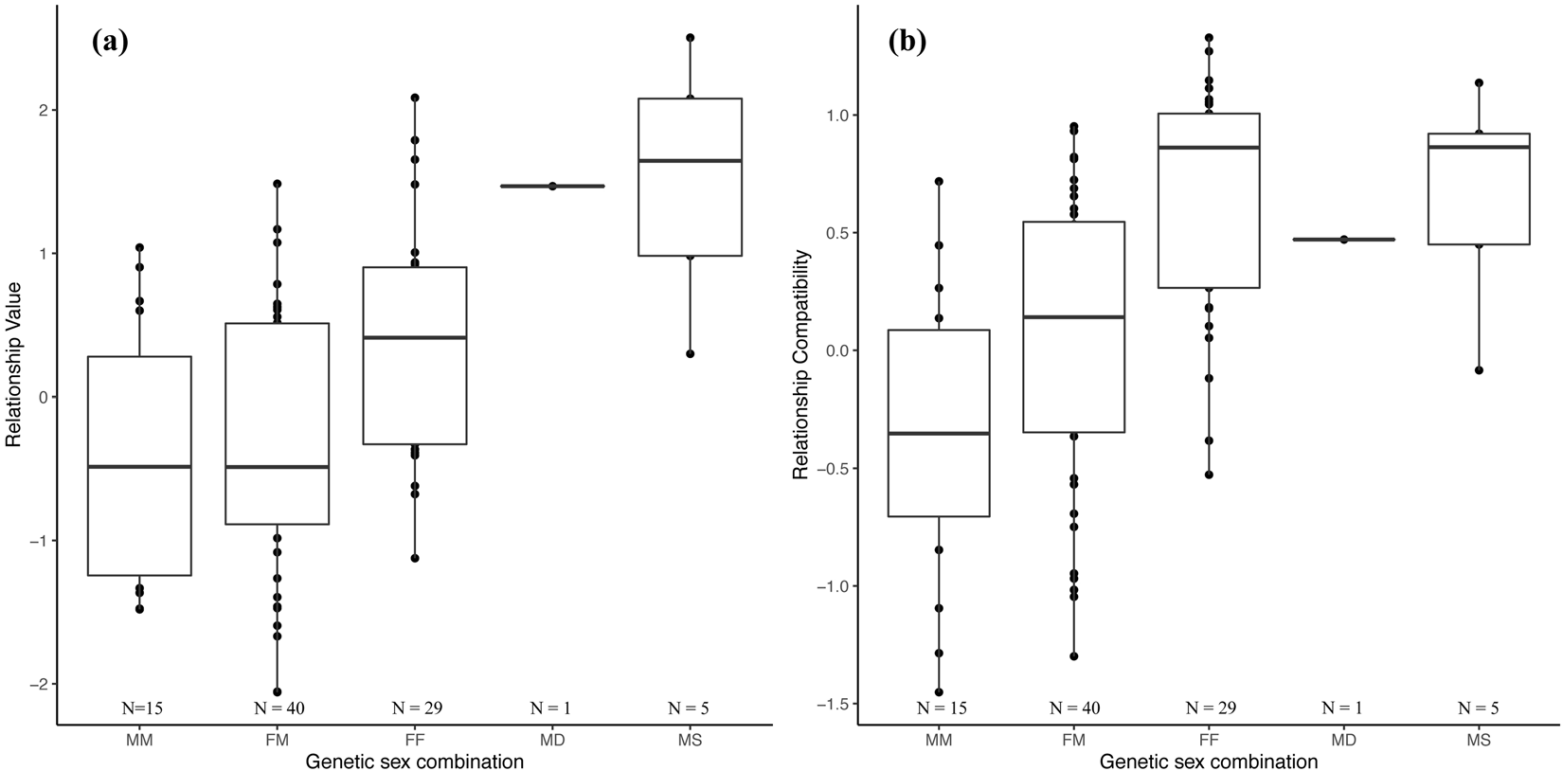
Mean (**a**) relationship value and (**b**) relationship compatibility of unrelated male-male (MM), unrelated female-male (FM), unrelated female-female (FF), mother-daughter (MD) and mother-son (MS) dyads.

Overall, the set of predictors significantly influenced relationship value (χ^2^ = 24.8, df = 7, *p* = 0.001). More specifically, relationship value differed substantially between the genetic sex combinations (χ^2^ = 15.8, df = 3, *p* = 0.001) (Fig. 1a), such that mother-son dyads had the highest relationship value (mean ± SD = 1.50 ± 0.88), followed by female-female dyads (mean ± SD = 0.40 ± 0.78), unrelated female-male dyads (mean ± SD = −0.33 ± 0.92), and male-male dyads (mean ± SD = −0.41 ± 0.89).

**Figure 2:**
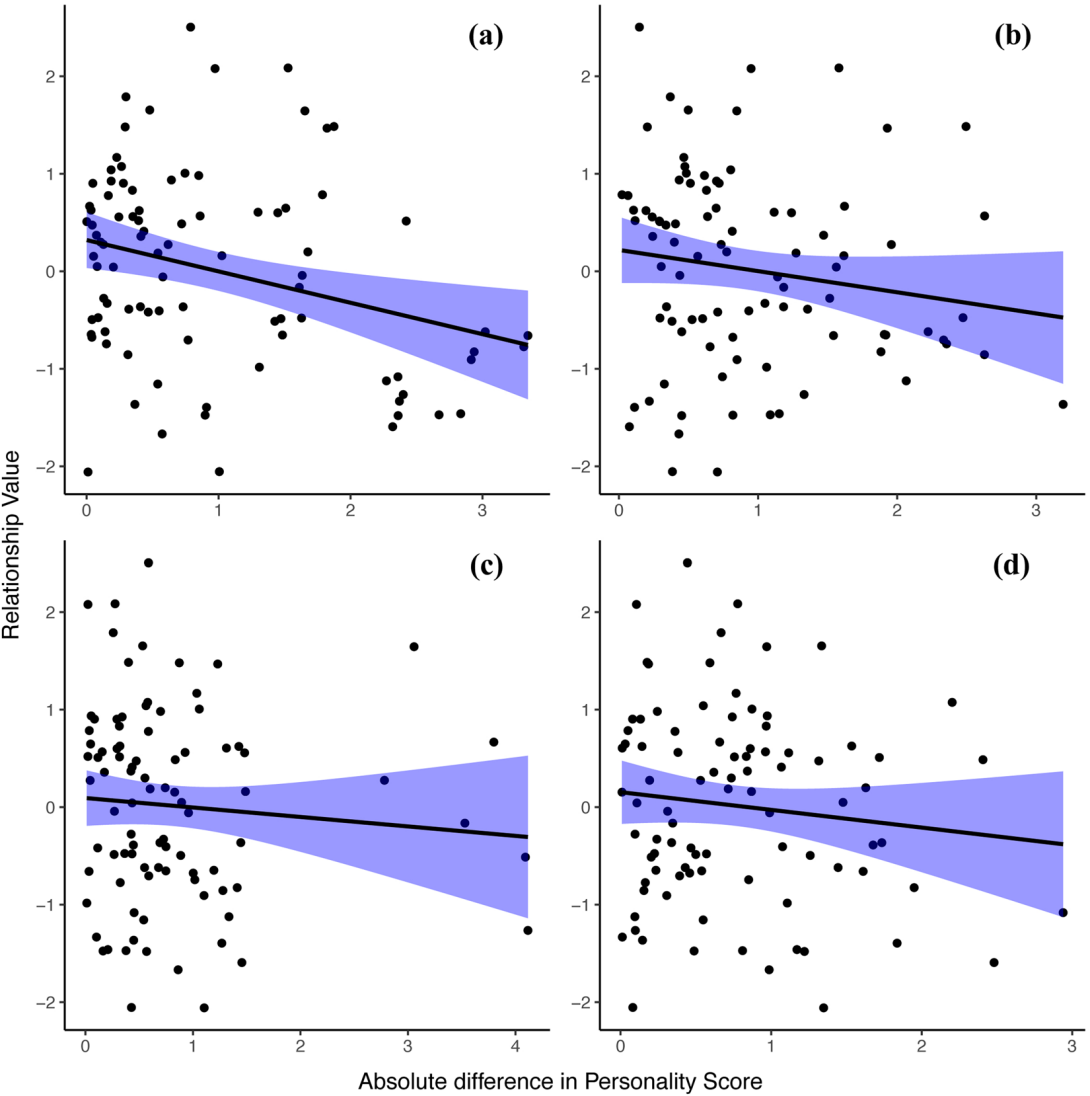
The link between relationship value and the absolute difference in personality score of (**a**) Sociability, (**b**) Openness, (**c**) Boldness and (**d**) Activity per dyad for all genetic sex combinations with corresponding confidence intervals.

Besides genetic sex combination, similarity in Sociability was also significantly, tough less apparent, associated with relationship value (χ^2^ = 4.1, df = 1, *p* = 0.042; Fig. 2a), with subjects having more similar Sociability scores exhibiting higher value relationships (estimate ± SD = −0.26 ± 0.09). The other personality traits did not significantly influence relationship value (all *p* > 0.05) (Table 2, Fig. 2). Data simulations showed that large and medium-large estimates will be detected in this model with a probability of 1 and 0.80, respectively, indicating high power. The probability of detecting small effects was 0.49, indicating intermediate power. Type 1 error rates were within reasonable boundaries (0.067 and 0.083 for the two 0-value estimates; see Supplementary Materials).

**Table 2:** Factors influencing relationship value, assessed with a General Linear Mixed Model (GLMM).

Fixed variable	Num df	χ²	β ± SE	*t* value	p
Genetic sex combination	3	15.8	0.347 ± 0.202	1.72	**0.001**
FF vs MF			−0.707 ± 0.174	−4.07	**<0.001**
FF vs MM			−0.611 ± 0.226	−2.71	**0.002**
FF vs MS			0.634 ± 0.469	1.35	0.576
Similarity in Sociability	1	4.1	−0.257 ± 0.090	−2.85	**0.042**
Similarity in Openness	1	1.9	−0.191 ± 0.129	−1.47	0.164
Similarity in Boldness	1	2.1	−0.132 ± 0.083	−1.59	0.145
Similarity in Activity	1	0.1	0.026 ± 0.076	0.34	0.745

Bold typeface indicates significant p values at the level alpha < 0.05.

#### Relationship compatibility

**Figure 3:**
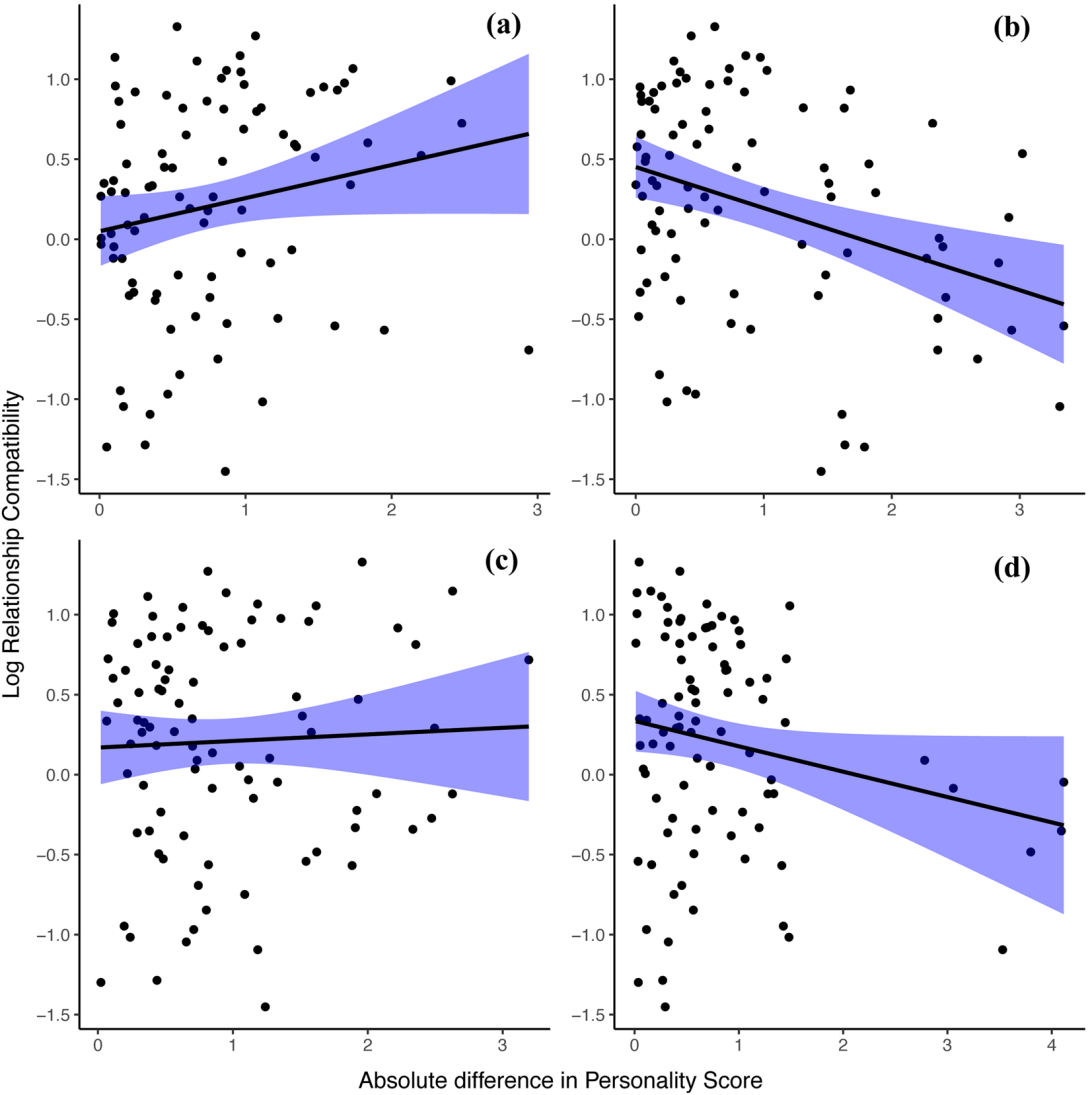
The link between relationship compatibility (log of the standardized scores) and the absolute difference in personality score of (**a**) Activity, (**b**) Sociability, (**c**) Openness, (**d**) Boldness per dyad for all genetic sex combinations with corresponding confidence intervals.

Overall, the set of predictors significantly influenced relationship compatibility (χ^2^ = 26.3, df = 7, *p* < 0.001). Relationship compatibility differed substantially between the genetic sex combinations (χ^2^ = 14.75, df = 3, *p* = 0.002, Fig. 1b). Mother-son dyads had the most compatible relationships (mean ± SD = 0.66 ± 0.48), followed by female-female dyads (mean ± SD = 0.64 ± 0.50) unrelated female-male dyads (mean ± SD = 0.04 ± 0.62), and male-male dyads (mean ± SD = −0.35 ± 0.63). Further, relationship compatibility was significantly associated with similarity in Activity (χ^2^ = 5.2, df = 1, *p* = 0.023; Fig. 3d): bonobos with relatively different Activity scores engaged in more compatible relationships (estimate ± SD = 0.20 ± 0.07) (Fig. 3). None of the other personality traits was associated with relationship compatibility (all *p* > 0.1, Table 3, Fig. 3). The data simulations showed that large and medium-large estimates will be detected in this model with a probability of 1 and 0.81, respectively, indicating high power. The probability of detecting small effects was 0.49, indicating intermediate power. Type 1 error rates were within reasonable boundaries (0.079 and 0.073 for the two 0-value estimates; see Supplementary materials).

**Table 3:** Factors influencing relationship compatibility, assessed with a General Linear Mixed Model (GLMM).

Fixed variable	Num df	χ²	β ± SE	*t* value	p
Genetic sex combination	3	14.8	0.496 ± 0.120	4.14	**0.002**
FF vs MF			−0.416 ± 0.154	−2.70	**<0.001**
FF vs MM			−0.692 ± 0.227	−3.04	**<0.001**
FF vs MS			0.494 ± 0.246	2.01	0.889
Similarity in Sociability	1	1.4	−0.112 ± 0.069	−1.63	0.241
Similarity in Openness	1	0.5	0.047 ± 0.057	0.82	0.485
Similarity in Boldness	1	2.2	−0.142 ± 0.086	−1.65	0.140
Similarity in Activity	1	5.2	0.199 ± 0.072	2.77	**0.023**

Bold typeface indicates significant p values at the level alpha < 0.05.

## Discussion

The general aim of this paper was to understand the role of kinship, sex and personality in shaping relationship quality of captive bonobos. Our results indicate that kinship and sex combination, as well as homophily in personality traits Sociability and Activity, affect relationship value and compatibility in bonobos.

Similar to the relationship quality model previously described^41^, our dimension reduction analysis revealed two components. Due to low item loadings of symmetry in affiliative behavior, the third factor, ‘relationship security’, was not retained in this study. Our first component of relationship quality, relationship value, is comparable to the first component in chimpanzees^36,38^, ravens^39^, Japanese macaques^40^, spider monkeys^54^, barbary macaques^43^, capuchin monkeys^31^, bonobos^41^ and dolphins^42^. This component was significantly influenced by genetic sex combination, with mother-son dyads showing the highest value. This is in line with bonobo socio-ecology, where mothers provide agonistic support to their (sub)adult sons against others^49,55,56^, enhance their mating success^57,58^ and show high levels of dyadic grooming^49^. Similarly, higher relationship values between kin were also previously described in chimpanzees^36,38^, ravens^39^, macaques^40^ and a previous bonobo study^41^. Unrelated female dyads also showed high relationship values, which is in line with higher frequencies of reciprocal support among them, even though they do not always spend more time in proximity and show lower levels of dyadic grooming^49^. In addition to genetic sex combination, relationship value was also significantly influenced by homophily in Sociability scores. Our Sociability dimension includes mainly affiliative behaviors (grooming frequency, density and diversity and the number of individuals). Interestingly, while bonobos with similarly high Sociability scores will need to be in proximity to behave affiliative, causing high value relationships, this homophily in Sociability effect also indicates that individuals with similarly low Sociability scores, likewise have high value relationships. Low Sociability individuals, who do not engage in many social interactions, therefore invest a lot in just a few social relations, resulting in rare, but high value relationships. Our Sociability dimension is comparable to the Sociability dimensions found in capuchin monkeys^31^ and chimpanzees^21^, where similarity in this personality trait also resulted in higher quality relationships with more dyadic affiliation^31^ and more contact-sitting^21^, respectively. The Sociability dimension most resembles the Extraversion dimension in humans^59,60^, who also prefer friends that are more similar in Extraversion scores^27,28^. We did not find homophily in any of the other personality traits for relationship value. Homophily in Openness resulted in high quality relationships in humans^27,61^ and capuchin monkeys^31^, but no such association was found in our study. Also for Boldness, we did not find any effect of similarity in Boldness on relationship value, while in chimpanzees^21^ and baboons^20^ dyads with more similar boldness scores showed more contact sitting and grooming, respectively. Chimpanzees with more similar Grooming Equity scores also showed more contact sitting, but only among non-kin^21^. The Grooming Equity factor in this study, however, comprised both grooming density and grooming diversity, two behaviors that were included in our Sociability factor.

Our second relationship quality component, compatibility, was also influenced by genetic sex combination. Unsurprisingly, the highest compatibility scores were found between mother-son dyads followed by unrelated female-female dyads, female-male dyads and male-male dyads. Aggression is most common between males and from females to males but rarely happens between females or from males to females^55,62,63^. Also in chimpanzees^36,38^, ravens^39^, macaques^40^ and a previous bonobo study^41^ higher compatibilities were found between related individuals. Relationship compatibility was also significantly influenced by personality, albeit less clear given the high spread of our data. Similarity in Activity resulted in lower compatibility scores meaning that individuals with similar Activity scores engage in more counter-interventions against each other and behave more aggressively against one another. Our Activity trait had a high positive loading for activity and a negative one for self-scratching. In addition, grooming density, and time spent in proximity to the leopard had loadings > |0.4| on Activity but were attributed to Sociability and Boldness, respectively, due to higher loadings on these factors.

In chimpanzees, activity and self-scratching loaded on two separate personality factors: Activity and Anxiety^21,64^. Similarity in these personality traits resulted in stronger friendships in unrelated chimpanzees^21^, while similar Activity levels in bonobos here result in less compatible relationships. This effect might partly be explained by an underlying sex bias in Activity scores. Additional analyses for sex effects on bonobo personality dimensions (see Supplementary materials) indicate that bonobo males score significantly higher on Activity than females. In chimpanzees, higher levels of self-directed behaviors in males, have been suggested to reflect the stress of their male dominated society^64^. Considering that female bonobos occupy the higher ranks^65,66^, our results are in line with potential dominance-related influences on personality. However, further studies are needed to confirm the link between Activity, self-scratching and rank. If these effects are present, dyads with more similar Activity scores and small rank differences would show higher dyadic frequencies of aggression and therefore have less compatible relationships. However, these effects are not linear, as shown by the high distribution of data points on the graph. Similarity in Sociability, Boldness and Openness did not influence relationship compatibility in our study.

While our bonobo personality factors, based on behavioral observations, are comparable to the personality factors in humans^26,27^ and chimpanzees^21^ different results concerning the effect of personality on friendships were found. One apparent explanation is that we implemented a different and perhaps more inclusive composite model to measure relationship quality^35^. In chimpanzees^21^, contact-sitting was used as a simple measure for friendship, while in humans, questionnaire answers were used instead of behavioral observations to determine relationship quality^27^. Studying the influence of personality on the composite measure for relationship quality in chimpanzees^36^ might be an interesting next step to further our understanding of the evolution of homophily in friendships in these two closely related species.

While the relationship between personality and friendship is clear in several species, less is known about its underlying mechanism. Do individuals choose others with similar personalities to form friendships or do personalities of individuals become more similar over time due to shared experiences? This attraction and/or convergence comparison^31^ requires a long-term study to compare personalities and relationship quality at consecutive points in time. Further, the role of personality in friendships seems to be trait-specific, as opposed to all traits being similar between friends, and the importance of different traits appears to be species-specific. Further research is therefore needed to study which benefits result from similarity in certain personality traits and whether the evolutionary fitness of dyads with similar personalities is higher than dissimilar dyads in both captive and wild populations.

In conclusion, we found that the quality of social bonds between bonobos is influenced by the genetic sex combination of both partners and their personality similarity, more specifically in Sociability and Activity. Homophily in Sociability is likely to be a shared feature in ourselves and our closest relatives, chimpanzees and bonobos. While similarity in Sociability might promote reliable high quality relationships through reciprocity in similarly affective behavioral tendencies, lower compatibility levels of dyads with more similar Activity scores may be a byproduct of rank differences.

## Methods

Behavioral data were collected for captive bonobos housed in six zoological parks: Planckendael (PL) in Mechelen, Belgium; Apenheul (AP) in Apeldoorn, the Netherlands; Twycross Zoo World Primate Centre (TW), Twycross, United Kingdom; Wuppertal Zoo (WU), Wuppertal, Germany; Frankfurt Zoo (FR), Frankfurt, Germany; and Wilhelma Zoological and Botanical Garden (WI) in Stuttgart, Germany. The subjects included 23 female and 16 male bonobos whose ages ranged from 7 to 63 years. All subjects were housed in groups that included juveniles and/or infants, which were excluded from the behavioral data collection. Behavioral data for relationship quality and personality analysis were collected during the same observational periods. Details on group composition and data collection can be found in the Supplementary Table S1.

### Measures and analysis

We collected a total of 1442.39 hours of focal observations (mean 16.37 hours per individual), 43506 group scans (mean 545 per individual) and 430.96 h of all occurrence observations during feedings (mean 28.73 hours per group). Inter-observer reliabilities reached a mean of r = 0.87 across all observers, meaning that all observations were highly reliable^67^. Live scoring of behavioral data was done using The Observer (Noldus version XT 10, the Netherlands).

### Personality profiles

Individual personality profiles were available and based on the personality model described in a previous paper^52^. The behavioral variables used to construct this model were derived from both naturalistic and experimental settings^52,68^. In short, we included a total of 17 behavioral variables (10 from the naturalistic context and 7 from the experimental contexts). Raw variables were standardized into z-scores for each group before combining data from different zoos. As the definition of personality requires stability of traits between individuals across time, data were collected in two consecutive years for each group allowing us to test for temporal consistency. Intraclass correlations were used to determine temporal stability and only variables that were stable were used to determine personality structure. Dimension reduction analysis on these variables revealed four factors: Sociability, Boldness, Openness and Activity^52^. Details of each item’s loading onto each dimension are shown in Table 4 (See also Supplementary Table S3). Items that showed cross-loadings > |0.4| on multiple components, were considered part of the dimension on which they had the highest loading.

### Relationship quality

Measures for relationship quality were determined based on the relationship quality model described in a previous paper on bonobos^41^. We extracted dyadic scores for 8 social behavioral variables, which were collected in a naturalistic setting: Aggression frequency, aggression symmetry, counter-intervention, grooming frequency, grooming symmetry, peering frequency, proximity, support (For definitions see: Supplementary Table S2). We then performed exploratory factor analysis (EFA) with varimax rotation and Kaiser normalization to extract composite measures for these 8 variables. The number of dimensions to extract was determined by inspecting the scree plot and by conducting a parallel analysis^69,70^. The factors were then subjected to a varimax rotation and variable loadings ≥ |0.4| were interpreted as salient.

### Linear mixed models

To determine potential associations between relatedness, sex combination, personality profiles and relationship quality measures, we used General Linear Mixed Models with Gaussian error distribution and identity link function (lme4 package 1.1-13^71^) for a total of 90 dyads. Rank difference was not included in our models to reduce the amount of overfitting. Similarity in personality per dyad was determined taking the absolute difference of the personality scores of both individuals of a dyad. The relationship quality components were treated as response variables in two different models. The full models comprised the different personality similarity variables (all *z*-transformed) and the fixed categorical variable “genetic sex combination” (denoting the demographic nature of the dyad: female-female, female-male, male-male, mother-daughter, mother-son) as predictor variables. Combining relatedness and sex combination in one factor (genetic sex combination) allows us to separate related female-male (mother-son dyads) from unrelated female-male dyads and compare results between them. Only one mother-daughter dyad was included in our sample and was therefore excluded from statistical analyses. The random effects structure consisted of intercepts for each of the two subjects in the dyad and for the location of observation (zoo), including the random slopes of the four personality variables within the subjects and zoo, and the additional random slopes of genetic sex combination (dummy coded) within zoo^72,73^. The null model was an intercept-only model, with the same random effects structure as the full model. Given the high number of estimated parameters in relation to the sample sizes (i.e., slight overfitting), we performed simulations to assess the power of our models. Data were corrected for observation time and diagnostic plots (residuals vs. fitted, QQ plots) were used to confirm the assumptions of normality and homogeneity of variances. When any of the assumptions were not met, we used square root, z- or log transformations of our variables. All statistical analyses were conducted using IBM SPSS Statistics 20 and R (version 3.4.3; R Core Team, 2017).

**Table 4:** The behavioral contents of the coded personality dimensions^52^.

Factor	Adjectives loading on to factor
Sociability	+Grooming frequencies + Grooming density + Neighbors + Grooming diversity − Latency to approach puzzles/durian − autogroom
Openness	+Approaches to puzzles/others + Play + Proximity to puzzles + Taste pasta (− Latency to approach Puzzle)
Boldness	+Approaches to leopard + displays to leopard + proximity to leopard + Aggression received
Activity	Self-scratching + Activity (+Grooming density given – Time in Proximity to Leopard)

### Ethical statement

No animals were sacrified or sedated for the purpose of this study. This study was approved by the Scientific Advisory Board of the Royal Zoological Society of Antwerp and the University of Antwerp (Belgium), and endorsed by the European Breeding Program for bonobos. All research complied with the ASAB guidelines^74^.

## Supplementary information

Supplementary material
