# Supplementary material for: Bonobo personality predicts friendship
Source: Sci Rep. 2019 Dec 17;9:19245. doi: 10.1038/s41598-019-55884-3 (PMC6917795; doi:10.1038/s41598-019-55884-3)
Supplement: Supplementary file 1 — Supplementary material [file 41598_2019_55884_MOESM1_ESM.docx]

**Bonobo personality predicts friendship**

Jonas Verspeek, Nicky Staes, Edwin van Leeuwen, Marcel Eens & Jeroen MG Stevens

# Supplementary

##### *Table S1 – Details on group composition, time of behavioral data collection and observers*

|  | | | | | |  |
| --- | --- | --- | --- | --- | --- | --- |
|  | **Zoo** | **Adults and subadults** | **Juveniles** | Period | **Observers** | **Focal time per individual (mean ± SD )** |
| **Round 1** | PL | 3M / 2F | 1M / 1F | Nov. 2011 - Jan. 2012 | AP | 21.74 ± 0.30 |
|  | AP | 2M / 4F | 2M / 2F | Mar. - Apr. 2012 | AS, NS | 17.59 ± 0.34 |
|  | WI | 3M / 9F | 2M / 2F | May – Jul. 2013. | MB | 17.31 ± 0.21 |
|  | FR | 3M / 6F | 3M / 2F | Apr. - May 2012 | SR, NS | 12.98 ± 0.24 |
|  | WU | 3M / 3F | 3M | May. - Jun. 2012 | SR, NS | 15.38 ± 0.46 |
|  | TW | 3M / 6F | 1M / 2F | Sep. – Nov. 2012 | NS | 13.57 ± 0.24 |
| **Round 2** | PL | 4M / 3F | 1F | Nov. 2012 - Jan. 2013 | WR | 20.25 ± 0.1 |
|  | AP | 2M / 4F | 1M / 2F | Feb. - May 2013 | LJ | 14.53 ± 0.55 |
|  | WI* | 3M / 9F | 2M / 1F | Jul. - Aug. 2014 | JV | 13.96 ± 0.31 |
|  | FR | 3M / 7F | 3M / 3F | Feb. - Apr. 2014 | MW | 17.22 ± 0.21 |
|  | WU | 3M / 2F | 2M | Jan. - Mar. 2013 | WR | 30.27 ± 0.38 |
|  | TW* | 3M / 6F | 2M / 2F | Aug. – Oct. 2013 | MB | 16.15 ± 0.26 |
| PL = Planckendael, AP=Apenheul, WI=Wilhelma Zoological and Botanical Garden, FR=Frankfurt Zoo, WU=Wuppertal Zoo, TW=Twycross Zoo World Primate Center. * In Twycross and Wilhelma collection of natural observations but no experimental data was done in round 2 | | | | | | |

##### *Table S2 - Behavioral variables, with the corresponding definitions, scored during the naturalistic observations to determine Relationship Quality (See Stevens et al.*, *2015).*

|  | |
| --- | --- |
| **Behavioral variable** | **Definition** |
| Grooming frequency | Number of grooming bouts exchanged within a dyad (i.e. the sum of all bouts from A to B and from B to A) |
| Grooming symmetry | Symmetry of grooming within a dyad A and B was calculated using the following formula: A grooms B/(A grooms B + B grooms A). For each dyad, the lowest of the two values obtained reversing A’s and B’s roles was chosen to represent the degree of symmetry (ranging from 0 to 0.5) ^+^. |
| Aggression frequency | Frequency of all aggressive interactions within a dyad |
| Aggression symmetry | Symmetry of aggression within a dyad (calculated in the same way as grooming symmetry). |
| Support | Index of agonistic support (frequency of support/opportunity to support). Support was defined as all instances where an individual A intervenes with an aggression within 30s in an agonistic interaction between two other individuals B and C to aid in attack or in defense. |
| Counter-intervention | Index of counter-intervention (frequency of counter-intervention/ opportunity to intervene). Every support pro for an individual implied a contra support against a victim. The target of a coalition is considered as the receiver of ‘counter-intervention’. |
| Peering frequency | Frequency of peering (i.e., “the actor stares at the receiver’s face from very close distance, up to a few centimeters”*) |
| Proximity | Proportion of scans spent within arm’s reach |
| ^+^ Fraser *et al*.; 2008; * Kano, 1992 | |

##### *Table S3 – Variable loadings dimension reduction personality model (from Staes et al., 2016)*

|  | Factor | | | |  |
| --- | --- | --- | --- | --- | --- |
| Variable | Sociability | Openness | Boldness | Activity | *h²* |
| Grooming Received | **0.83** | -0.03 | -0.03 | 0.20 | 0.74 |
| Grooming Density Received | **0.76** | -0.07 | -0.12 | 0.14 | 0.68 |
| Number of Neighbors | **0.71** | 0.13 | 0.13 | -0.04 | 0.54 |
| Grooming Given | **0.67** | 0.18 | 0.13 | 0.39 | 0.69 |
| Latency to Approach Puzzle | **-0.66** | **-0.49** | 0.02 | 0.24 | 0.79 |
| Grooming Density Given | **0.64** | 0.20 | 0.33 | **0.42** | 0.84 |
| Latency to Approach Durian | **-0.64** | -0.23 | -0.01 | 0.14 | 0.47 |
| Grooming Diversity Index | **0.53** | 0.12 | 0.19 | 0.36 | 0.67 |
| Autogroom | **-0.48** | 0.10 | -0.39 | 0.01 | 0.46 |
| Puzzle Number of Approaches | 0.08 | **0.91** | 0.13 | 0.06 | 0.83 |
| Play | -0.07 | **0.70** | 0.00 | 0.22 | 0.63 |
| Time in Proximity to Puzzle | 0.20 | **0.68** | -0.31 | 0.03 | 0.59 |
| Approach others | 0.05 | **0.65** | 0.35 | 0.27 | 0.69 |
| Taste Pasta | 0.27 | **0.41** | 0.20 | 0.11 | 0.42 |
| Leopard Number of Approaches | 0.02 | 0.11 | **0.82** | 0.02 | 0.67 |
| Leopard Number of Displays | 0.21 | 0.07 | **0.62** | -0.01 | 0.48 |
| Time in Proximity to Leopard | 0.10 | -0.08 | **0.59** | **-0.44** | 0.54 |
| Aggression Received | -0.37 | 0.12 | **0.54** | 0.31 | 0.54 |
| Scratch | -0.10 | -0.17 | 0.19 | **-0.69** | 0.66 |
| Activity | 0.29 | 0.30 | 0.26 | **0.53** | 0.65 |
| Eigenvalue | 5.98 | 2.85 | 2.59 | 1.73 |  |
| % variance explained | 29.92 | 14.25 | 12.93 | 8.65 |  |
| Boldface indicates loadings >\|0.40\| | | | | | |

##### *Supplementary information about the data simulations to assess the power of our models*

Given that our models were slightly over-fitted (i.e., *n*=89, parameters assessed: 27), possibly leading to unstable results and low power, we assessed the stability and power of the models by *i*) by excluding subjects one at a time from the data and comparing the model estimates derived for these data with those derived for the full data set (indicating no influential subjects to exist), and *ii*) simulating new instances of the response and computing the frequencies by which simulated large, medium, small and 0-value fixed effects estimates reached significance for the full-null model comparison as well as for individual effects.. For each model, we ran 1,000 simulations (script by Roger Mundry, available upon request). The magnitude of the simulated random effects we chose based on the model fitted to the original data (0.2 for the random intercepts and slopes of the two individuals; 0.5 for the random intercept of zoo, and 0 for the random slopes within zoo). If the simulated models, consisting of the same fixed and random effect structures as the original models, yielded high probability of obtaining a significant result for large and medium estimates (power > 0.7), and low probability of obtaining a significant result for 0-value estimates (Type 1 error rate < 0.1), we considered our models to be sufficiently stable for drawing valid inferences.

*Supplementary analyses to test sex and age associations with personality*

Sex and age effects on personality dimensions were tested using linear mixed models. Sex and age were entered as fixed effects. Group was entered as a random intercept to account for non-independence of observations within the same zoo. The significance of fixed effects was tested using an F-test with a Kenward-Roger correction for the number of degrees of freedom. The F-test was performed using the add-on package pbkrtest (Halekoh & Hojsgaard, 2014). Female bonobos scored significantly higher than males on Openness (F(1,35)=7.70, p=0.009), but lower on Activity (F(1,36)=6.63, p=0.014). They did not differ from males in Sociability F(1,36)=2.68, p=0.110) and Boldness scores F(1,38)=2.34, p=0.134). An age effect was found for Openness, with younger bonobos scoring higher on this dimension (F(1,36)=25.51, p<0.001, β=-0.05). No significant age effects were found for the other dimensions (Sociability F(1,34)=0.78, p=0.382; Boldness F(1,37)=1.65, p=0.201; Activity F(1,35)=1.77, p=0.192).
